# Multicriteria Personnel Selection by the Modified Fuzzy VIKOR Method

**DOI:** 10.1155/2015/612767

**Published:** 2015-09-30

**Authors:** Rasim M. Alguliyev, Ramiz M. Aliguliyev, Rasmiyya S. Mahmudova

**Affiliations:** Institute of Information Technology, Azerbaijan National Academy of Sciences, 9 B. Vahabzade Street, AZ1141 Baku, Azerbaijan

## Abstract

Personnel evaluation is an important process in human resource management. The multicriteria nature and the presence of both qualitative and quantitative factors make it considerably more complex. In this study, a fuzzy hybrid multicriteria decision-making (MCDM) model is proposed to personnel evaluation. This model solves personnel evaluation problem in a fuzzy environment where both criteria and weights could be fuzzy sets. The triangular fuzzy numbers are used to evaluate the suitability of personnel and the approximate reasoning of linguistic values. For evaluation, we have selected five information culture criteria. The weights of the criteria were calculated using worst-case method. After that, modified fuzzy VIKOR is proposed to rank the alternatives. The outcome of this research is ranking and selecting best alternative with the help of fuzzy VIKOR and modified fuzzy VIKOR techniques. A comparative analysis of results by fuzzy VIKOR and modified fuzzy VIKOR methods is presented. Experiments showed that the proposed modified fuzzy VIKOR method has some advantages over fuzzy VIKOR method. Firstly, from a computational complexity point of view, the presented model is effective. Secondly, compared to fuzzy VIKOR method, it has high acceptable advantage compared to fuzzy VIKOR method.

## 1. Introduction

Personnel evaluation is a complex process in the scope of which many factors should be evaluated simultaneously in a decision-making process. Evaluation process should provide reliable and valid information about alternatives. There are some conventional techniques used in this process: mainly, completion of application forms, initial interview, employment test, and background investigation. The conventional personnel evaluation techniques that are developed on the basis of static job characteristics will no longer suffice. These methods generally come to a conclusion on the basis of the subjective judgment of decision-maker, which makes the accuracy of the results highly questionable. Moreover, these methods take into consideration some classical criteria (age, experience, etc.) in the decision-making process [1, 2]. Various studies have been conducted on personnel evaluation problem to eliminate the drawbacks of conventional personnel evaluation techniques [3–7]. Chien and Chen [3] reviewed the personnel evaluation studies and found that the important issues including change in organizations, change in work, change in personnel, change in the society, change of laws, and change in marketing have influenced personnel evaluation and recruiting. Personnel recruitment and evaluation directly affect the quality of employees [3]. Hence, various new technologies, like computer-based testing, internet-based testing, telephone-based interviews, video-conference job interviews, and multimedia simulation tests, allow organizations to test large numbers of applicants at the same time and help in saving time and money and make better personnel evaluation decisions [8].

Recent studies stated that an information culture plays an important role in the success of the modern organizations [9–14]. Information culture is an important factor that must be stimulated in all types of modern organization management. Khan and Azmi [15] explained that information culture is a culture where information is the essence of all activities in organization. Choo et al. [16] and Bergeron et al. [17] looked at information culture as the socially shared patterns of behaviors, norms, and values that define the significance and use of information in an organization. To profile an organization's information culture, in more recent studies, Choo et al. [18] adapted six criteria: (1)* information integrity*, (2)* information formality*, (3)* information control*, (4)* information transparency*, (5)* information sharing*, and (6)* information proactiveness*. Choo [9] presented four information culture types as follows: result-oriented: it pursues goal achievement and competitive advantage; rule-following: it pursues control, compliance, and accountability; relationship-based: it encourages communication, participation, and commitment; risk-taking: it encourages innovation, creativity, and exploration of new ideas. Each information culture type may be characterized by a set of 5 attributes: the primary goal of information management, information values and norms, information behaviors in terms of information needs, information seeking, and information use. Authors of [19] state that information culture of personnel may be characterized by a set of five criteria, namely, (1)* information gathering and perception skill*; (2)* information memorization skill*; (3)* information handling skill*; (4)* information protection and security skill*; and (5)* information presentation skill*. So, in this study, for personnel evaluation, we will use these criteria which will be explained in Section 4.

It is known that selecting the best alternative among many alternatives is a multicriteria decision-making (MCDM) problem. MCDM is one of the most widely used decision methodologies in science, business, and engineering worlds. MCDM methods aim at improving the quality of decisions by making the process more explicit, rational, and efficient [20, 21]. A typical MCDM problem involves a number of alternatives to be evaluated and a number of criteria to evaluate the alternatives. MCDM methods deal with problems of compromise evaluation of the best solutions from the set of available alternatives according to objectives.

In this paper, an integrated fuzzy MCDM approach was proposed for the information personnel evaluation process. The objective of this study is to determine the evaluation criteria and to evaluate personnel by means of modified VIKOR [22] under a fuzzy environment. In this study, the “worst-case” method [23] is used to determine the relative weight of the criteria and the modified fuzzy VIKOR method is proposed to rank the alternatives in terms of overall performance with respect to multiple information culture criteria.

This paper is organized as follows. In Section 2, we first introduce the literature review on MCDM in personnel evaluation.  Section 3 describes basic concepts of fuzzy sets and fuzzy numbers. In Section 4, a modified fuzzy VIKOR method is presented and its steps are determined in detail.  Section 5 describes worst-case method, which is used to determine weights of the evaluation criteria. How the proposed model is used on a real world example is explained in Section 6. Finally, we conclude this paper in Section 7.

## 2. Literature Review on MCDM in Personnel Evaluation

Among the MCDM problems that we encounter in real life is the personnel evaluation problem. The personnel evaluation problem, from the multicriteria perspective, has attracted the interest of many researchers. In the literature, there are a number of studies that have been conducted on personnel evaluation. The world around us is difficult to see in one-dimensional way in order to assess what we see. So we always compare and rank objects of our choice with respect to different criteria. In this context MCDM is a powerful tool widely used in evaluating, selecting, or ranking a finite set of decision alternatives characterized by multiple and usually conflicting criteria [24–26]. It can help users understand the results of integrated assessments. At present, different researchers have proposed many MCDM methods: AHP (Analytic Hierarchy Process) [27, 28], TOPSIS (Technique for Order Preference by Similarity to Ideal Solution) [29], VIKOR (VIsekriterijumska Optimizacija i KOmpromisno Resenje: multicriteria optimization and compromise solution) [22], ELECTRE II (ELimination Et Choix Traduisant la REalité: ELimination and Choice Translating REality) [30], PROMETHEE II (Preference Ranking Organization METHod for Enrichment Evaluation) [31], and DEA (Data Envelopment Analysis) [32]. Due to vagueness in the data and ambiguity in decision-making process, fuzzy set theory has been incorporated into MCDM techniques by many researchers for personnel selection problem. Among these techniques, TOPSIS and VIKOR methods seemed to be more appropriate for solving the personnel selection problem because they have capability to deal with each kind of judgment criteria, having clarity of results and easiness to deal with attributes and decision options [33].

TOPSIS was proposed by Hwang and Yoon [29] to determine the best alternative based on the concepts of the compromise solution. The main concept of the TOPSIS method is that the chosen alternative should have the shortest Euclidean distance from the positive-ideal solution and the farthest Euclidean distance from the negative-ideal solution. Positive-ideal solution is the one that maximizes the benefit criteria and minimizes the cost criteria, while the negative-ideal solution maximizes the cost criteria and minimizes the benefit criteria. This method defines an index called closeness to positive-ideal solution and the farthest from the negative-ideal solution. Finally, this method chooses an alternative with the maximum closeness to the positive-ideal solution. The compromise solution can be regarded as choosing the solution with the shortest Euclidean distance from the ideal solution and the farthest Euclidean distance from the negative-ideal solution [33–36].

The AHP method requires pairwise comparison of various alternatives with respect to each of the criteria and pairwise comparison criteria themselves. When the number of alternatives and/or criteria increases then the size and number of the comparison matrices increase. The improved AHP method by eliminating the comparison matrices required for alternatives was suggested by Rao [36]. In the improved AHP method, by normalizing the values of criteria by a systematic way, the rank reversal problem also is removed. To avoid the laborious procedures of generating and processing paired comparison matrices, Rotshtein [23] proposes worst-case method. This method instead of pairwise comparison uses special relations based on comparison with the worst alternative and with the least important criterion.

The highest ranked alternative by TOPSIS is the best in terms of the ranking index, which does not mean that it is always closest to the positive-ideal solution. Hence a new approach based on fuzzy VIKOR method is also used to obtain the rankings. This method focuses on ranking and selecting from a set of alternatives and determines compromise solutions for a problem with conflicting criteria [33]. VIKOR is a helpful tool in MCDM, particularly in a situation where the decision-maker is not able or does not know how to express preference in the beginning of system design. VIKOR ranks alternatives and determines the solution named compromise that is the closest to the ideal. Su [37] proposed a hybrid fuzzy approach, which assesses each alternative in terms of distance measure calculated by a modified VIKOR method.

The AHP method has been criticized as decision problems are structured in a hierarchical manner. Some decision-making problems cannot be structured hierarchically because they involve the interaction and dependence of higher level elements on lower elements. To solve the problem of dependence among alternatives and criteria, Saaty [28] proposed the use of analytic network process (ANP). The ANP was proposed to extend the AHP to release the restrictions of the hierarchical structure, which indicates that the criteria are independent from each other.

AHP can effectively handle both qualitative and quantitative data, but the conventional AHP still cannot reflect the ambiguity in human thinking style. Therefore, to solve these problems, some solutions to fuzzy MCDM problems were proposed, such as fuzzy AHP and fuzzy TOPSIS. They are the key tools used to solve the evaluation and scheduling problems under the fuzzy multicriteria conditions [38]. Güngör et al. [39] have developed a personnel evaluation system based on fuzzy AHP. Dursun and Karsak [6] presented a fuzzy MCDM algorithm using the principles of fusion of fuzzy information, 2-tuple linguistic representation model, and TOPSIS. Lately, Zhang and Liu [5] and Guo [40] used grey relational analysis to solve the personnel evaluation problem under an intuitionistic fuzzy environment. In [3], a data mining framework for personnel evaluation to explore the association rules between personnel characteristics and work behaviors has been developed, including work performance and retention.

Numerous fuzzy MCDM methods have been developed and there is no best method for the general fuzzy MCDM problem. Most fuzzy number ranking methods suffer from various drawbacks such as (a) lack of sensitivity when comparing similar fuzzy numbers, (b) counterintuitive outcomes in certain circumstances, and (c) complex computational processes [20, 41]. Therefore, in recent years, researchers have attempted to combine different methods to select the best alternative. For supporting the personnel evaluation process in the manufacturing systems, Dağdeviren [2] proposed a hybrid model which employs ANP and modified TOPSIS. Lin [32] combined ANP with fuzzy data envelopment analysis and proposed an integrated method to solve the personnel evaluation problem. For solving a personnel evaluation problem Baležentis et al. [42] proposed the new hybrid MULTIMOORA-FG method to cope with group decision making by employing fuzzy weighted averaging operator. Further, in [43], the MULTIMOORA method was extended by employing type-2 fuzzy sets with generalized interval-valued trapezoidal fuzzy numbers. The new fuzzy MULTIMOORA method, as in the case of the crisp MULTIMOORA, consists of three parts, namely, the Ratio System, the Reference Point, and the Full Multiplicative Form, representing different approaches of data aggregation.

## 3. Basic Concepts of Fuzzy Sets and Fuzzy Numbers

Fuzzy set theory, introduced by Zadeh [44], deals with problems in which a source of vagueness is involved and has been utilized for incorporating imprecise data into the decision framework.

A tilde “~” will be placed above a symbol if the symbol represents a fuzzy set.

**Definition 1 (fuzzy set).**

Let *U* be the universe of discourse, *U* = {*u*_1_,…, *u*_*n*_}. A fuzzy set $\tilde{A}$ of *U* is a set of order pairs $\left\{(u_{1},\mu_{\tilde{A}}(u_{1})),\ldots ,(u_{n},\mu_{\tilde{A}}(u_{n}))\right\}$, where $\mu_{\tilde{A}}:U\to [0,1]$ is the membership function of $\tilde{A}$ and $\mu_{\tilde{A}}(u_{i})$ stands for the membership degree of *u*_*i*_ in $\tilde{A}$.

A fuzzy number is an extension of a regular number in the sense that it does not refer to one single value but rather to a connected set of possible values, where each possible value has its own weight between 0 and 1. This weight is called the membership function. In fact the use of triangular fuzzy numbers (TFNs) is easy and understandable for decision-makers. In this paper we also tool for the use them as a proper for the study.

**Definition 2 (fuzzy number).**

A TFN denoted by $\tilde{A}$ is defined as follows: $\tilde{A}=(a^{l},a^{m},a^{u})$, where *a* ^*l*^ ≤ *a* ^*m*^ ≤ *a* ^*u*^. *a* ^*l*^ denotes the smallest possible value, while *a* ^*m*^ is the most promising value, and *a* ^*u*^ is the largest possible value. Each TFN has linear representations on its left and right side such that its function can be defined as

(1) $$\begin{array}{c} \mu_{\tilde{A}}\left(\;x\;\right)=\left\{\begin{array}{cc} 0, & x<a^{l}\;or\;x>a^{u},\\ \frac{x-a^{l}}{a^{m}-a^{l}}, & a^{l}\leq x\leq a^{m},\\ \frac{a^{u}-x}{a^{u}-a^{m}}, & a^{m}\leq x\leq a^{u}. \end{array}\right. \end{array}$$

$\mu_{\tilde{A}}(a^{m})=1$ for *a* ^*m*^ to be on the top of the triangular fuzzy number.

Mathematical operations on TFNs are defined as follows.

**Definition 3 (operations).**

The following arithmetic operations can be performed on TFNs. Let $\tilde{A}=(a^{l},a^{m},a^{u})$ and $\tilde{B}=(b^{l},b^{m},b^{u})$ be two TFNs. Then fuzzy arithmetic operations are defined as follows.

*(i) Summation*. One has $\tilde{A}+\tilde{B}=(a^{l}+b^{l},a^{m}+b^{m},a^{u}+b^{u})$.

*Example  1*. Let $\tilde{A}=(2,5,7)$ and $\tilde{B}=(3,4,6)$ be two TFNs. Then

(2) $$\begin{array}{c} \tilde{A}+\tilde{B}=\left(\;2+3,5+4,7+6\;\right)=\left(\;5,9,13\;\right). \end{array}$$

*(ii) Subtraction*. One has $\tilde{A}-\tilde{B}=(a^{l}-b^{u},a^{m}-b^{m},a^{u}-b^{l})$.

*Example  2*. Let $\tilde{A}=(2,5,7)$ and $\tilde{B}=(3,4,6)$ be two TFNs. Then

(3) A~−B~2,5,7−3,4,6=2−6,5−4,7−3=−4,1,4.

*(iii) Multiplication*. Scalar multiplication of TFN by a real number is as follows:

(4) $$\begin{array}{c} r\tilde{A}=\left\{\begin{array}{cc} \left(ra^{l},ra^{m},ra^{u}\right), & \text{if }r\geq 0,\\ \left(ra^{u},ra^{m},ra^{l}\right), & \text{if }r<0, \end{array}\right. \end{array}$$

where *r* denotes a real number.

*Example  3*. Let $\tilde{A}=(-2,4,9)$ be a TFN and *r* = 3. Then

(5) r×A~3×−2,4,9=3×−2,3×4,3×9=−6,12,27.

*Example  4*. Let $\tilde{A}=(-2,4,9)$ be a TFN and *r* = −3. Then

(6) r×A~−3×−2,4,9=−3×9,−3×4,−3×−2=−27,−12,6.

Multiplication of two TFNs is as follows:

(7) A~×B~=min⁡al×bl,al×bu,au×bl,au×bu,am×bm,max⁡al×bl,al×bu,au×bl,au×bu.

*Example  5*. Let $\tilde{A}=(2,5,7)$ and $\tilde{B}=(3,4,6)$ be two TFNs. Then

(8) A~×B~=min⁡2×3,2×6,7×3,7×6,5×4,max⁡2×3,2×6,7×3,7×6=6,20,42.

*(iv) Division. *Consider

(9) A~÷B~=min⁡al÷bl,al÷bu,au÷bl,au÷bu,am÷bm,max⁡al÷bl,al÷bu,au÷bl,au÷bu.

*Example  6*. Let $\tilde{A}=(2,5,7)$ and $\tilde{B}=(3,4,6)$ be two TFNs. Then

(10) A~÷B~min⁡23,26,73,76,54,max⁡23,26,73,76=26,54,73=13,54,73.

*(v) The Fuzzy Inverse. *One has

(11) $$\begin{array}{c} {\left(\;\tilde{A}\;\right)}^{-1}=\left(\;\frac{1}{a^{u}},\frac{1}{a^{m}},\frac{1}{a^{l}}\;\right). \end{array}$$

*Example* *7*. Let $\tilde{A}=(2,5,7)$; then ${\left(\tilde{A}\right)}^{-1}=\left(1/7,1/5,1/2\right)$.

*(vi) Max and Min.* Consider

(12) max⁡A~,B~=max⁡al,bl,max⁡am,bm,max⁡au,bu,min⁡A~,B~=min⁡al,bl,min⁡am,bm,min⁡au,bu.

*Example  8*. Let $\tilde{A}=(2,5,7)$ and $\tilde{B}=(3,4,6)$ be two TFNs. Then

(13) max⁡A~,B~max⁡2,3,max⁡5,4,max⁡7,6=3,5,7,min⁡A~,B~min⁡2,3,min⁡5,4,min⁡7,6=2,4,6.

*(vii) The Absolute Value*. The absolute value of the TFN $\tilde{A}=(a^{l},a^{m},a^{u})$ is denoted by $\left|\tilde{A}\right|$ and defined as follows [45–47]:

(14) $$\begin{array}{c} \left|\;\tilde{A}\;\right|\left(\;x\;\right)=\left\{\begin{array}{cc} \max\left\{\tilde{A}\left(x\right),-\tilde{A}\left(-x\right)\right\}, & \text{if }x\geq 0,\\ 0, & \text{if }x<0. \end{array}\right. \end{array}$$

*Example  9*. Let us consider $\tilde{A}=(4,7,11)$. Since *a* ^*l*^ = 4 > 0, then

(15) $$\begin{array}{c} \left|\;\tilde{A}\;\right|=\tilde{A}=\left(\;4,7,11\;\right). \end{array}$$

*Example  10*. Let us consider $\tilde{A}=(-8,-5,-1)$, so $-\tilde{A}=(1,5,8)$. Since *a* ^*u*^ = −1 < 0, then

(16) $$\begin{array}{c} \left|\;\tilde{A}\;\right|=-\tilde{A}=\left(\;1,5,8\;\right). \end{array}$$

*Example  11*. Let $\tilde{A}=(-2,4,6)$, so $-\tilde{A}=(-6,-4,2)$. Since 0 ∈ [*a* ^*l*^, *a* ^*u*^] = [−2,6], then

(17) $$\begin{array}{c} \left|\;\tilde{A}\;\right|=\left(\;0,4,6\;\right). \end{array}$$

*Example  12*. Let us consider $\tilde{A}=(-8,3,6)$, so $-\tilde{A}=(-6,-3,8)$. Since 0 ∈ [*a* ^*l*^, *a* ^*u*^] = [−8,6], then

(18) $$\begin{array}{c} \left|\;\tilde{A}\;\right|=\left(\;0,3,8\;\right). \end{array}$$

**Definition 4 (distance).**

Let $\tilde{A}=(a^{l},a^{m},a^{u})$ and $\tilde{B}=(b^{l},b^{m},b^{u})$ be two TFNs. The distance between fuzzy numbers $\tilde{A}$ and $\tilde{B}$ is calculated as

(19) dA~,B~=13al−bl2+am−bm2+au−bu2.

*Example  13*. Let $\tilde{A}=(-1,5,10)$ and $\tilde{B}=(3,8,14)$ be two TFNs. Then

(20) dA~,B~13−1−32+5−82+10−142=1316+9+16=413≈3.7.

**Definition 5 (aggregation).**

Assume that a decision group has *K* decision-makers DM_*k*_ (*k* = 1,2,…, *K*) and the fuzzy rating of each decision-maker can be represented as a positive TFN ${\tilde{A}}_{k}=(a_{k}^{l},a_{k}^{m},a_{k}^{u})$ with membership function $\mu_{{\tilde{A}}_{k}}(x)$. Then the aggregated fuzzy rating $\tilde{A}=(a^{l},a^{m},a^{u})$ can be defined as follows:

(21) al=1K∑k=1Kakl,am=1K∑k=1Kakm,au=1K∑k=1Kaku.

*Example  14*. Let ${\tilde{A}}_{1}=(-2,4,11)$, ${\tilde{A}}_{2}=(-3,2,7)$, ${\tilde{A}}_{3}=(2,6,9)$, and ${\tilde{A}}_{4}=(4,6,8)$ be four TFNs. Then the aggregated fuzzy rating $\tilde{A}=(a^{l},a^{m},a^{u})$ is

(22) al−2−3+2+44=0.25;am4+2+6+64=4.5;au11+7+9+84=8.75.

So $\tilde{A}=(0.25,4.5,8.75)$.

**Definition 6 (defuzzification).**

The defuzzification value of $\tilde{A}$ is an approximated real number. Various defuzzification strategies are suggested, while centroid method (often called center of area) is the most prevalent and physically appealing of all the defuzzification methods [48, 49]. In this study the fuzzy numbers are defuzzified using centroid method. For the triangular fuzzy numbers, $\tilde{A}=(a^{l},a^{m},a^{u})$, the defuzzified value is

(23) $$\begin{array}{c} A=\frac{\left(a^{l}+a^{m}+a^{u}\right)}{3}. \end{array}$$

*Example  15*. Let us consider $\tilde{A}=(-3,2,11)$. Then the defuzzification value of $\tilde{A}$ is *A* = (−3 + 2 + 11)/3 = 3.33.

## 4. The Modified Fuzzy VIKOR Method

The VIKOR method was developed for multicriteria optimization of complex systems. This method focuses on ranking and selecting from a set of alternatives and determines compromise solutions for a problem with conflicting criteria, which can help the decision-makers to reach a final decision [50–52]. This method determines the compromise solution and is able to establish the stability of decision performance by replacing the compromise solution obtained with initial weights [53]. An extension of VIKOR to determine fuzzy compromise solution for multicriteria is presented in [54]. The fuzzy VIKOR method has been developed to solve problem in a fuzzy environment where both criteria and weights could be fuzzy sets [52]. In this study, the TFNs are used to handle imprecise numerical quantities. Fuzzy VIKOR is based on the aggregating fuzzy merit that represents distance of an alternative to the ideal solution. The fuzzy operations and procedures for ranking fuzzy numbers are used in developing the fuzzy VIKOR algorithm.

In VIKOR, each alternative is measured based on an aggregate function, so the compromise ranking of alternatives is implemented by comparing the measure of closeness to the ideal solution. One of the characteristics of VIKOR is that always aggregate function is closest to the best solutions, while in TOPSIS the closeness indices of alternatives necessarily are not always very close to the ideal values. The fuzzy VIKOR procedure consists of the following steps [22, 53–55].

**Step 1 (identification of necessary criteria for personnel evaluation).**

In this step, the criteria that will be used to evaluate personnel are described briefly. Compared with the previous studies, the criteria for evaluating personnel are identified through literature review and investigation. The criteria that will be used to evaluate personnel are briefly explained as follows.

*Culture of Gathering and Perception of Information (C*_1_). Information gathering is not limited to passive gather of only provided information by a person. It is a process that starts with comprehension of information demand necessary for solving any problem and combines skills such as information processing on existing information resources and their structure, knowledge of information search algorithms in different information sources, determination of list of terms and key words in subjects, use of traditional and electronic information retrieval engines, and comprehension of obtained information regardless of presentation form and its types (text, audio, video, scheme, graph, etc.).

*Culture Memorization of Information (C*_2_). Information memorization is one of the important components of information culture of an individual. Memorization process is connected to characteristics such as human memory and attention. Memory has a significant role and importance for succeeding in life. According to scientists, memory is the ability of a person to store obtained knowledge and experience and use them during his/her life and activities.

*Culture Handling of Information (C*_3_). New skills are required from people in the period of rapid changes, occurring in society, and fast growth of information knowledge. A person must be able to analyze, evaluate information related to his activity, and create new information. Otherwise, it will be difficult to use obtained information and knowledge during the decision-making process regarding a certain issue. As along with abundance of information, availability of needless, uncertain information causes confusion. Thus, ability to evaluate, select, and analyze information is significant for professional activity of person.

*Culture Protection and Security of Information (C*_4_). New value of information and its conversion into strategic resource brings its protection and provision of its safety to the foreground in modern period. Solving the information security problem depends on not only technical methods and devices, but also culture of people. Currently, ability to implement the processes of collection, storage, processing, and transfer of information using computer, Internet, and other technical devices increases the risk of interception of confidential information by others. Information confidentiality problem is one of the main conditions of provision of information security. People working with information resources must comprehend their responsibility to protect the confidentiality of information belonging to different citizens or organizations.

*Culture Presentation of Information (C*_5_). A person demonstrates possession of rich knowledge, valuable information, when he/she can present carried knowledge and information to society. Information presentation is usually carried out through oral speech and writing, each of which has its own characteristics. The person mastering these characteristics well can present information on a perfect level. For example, information presentation skills of a person can be highly evaluated, when information is clear to those being presented to; that is, it is composed of simple and substantial sentences, excessive use of special terms being avoided, and carried ideas which are consecutive and complete each other. At the same time, field related to the presented information must be well researched, analyzed, and referred to when necessary.

**Step 2 (establish a group of decision-makers).**

Let *A*_*i*_ (*i* = 1,…, *n*) be a finite set of *n* alternatives which are to be evaluated by a group of *K* decision-makers DM_*k*_ (*k* = 1,…, *K*) with respect to a set of *m* evaluation criteria *C*_*j*_ (*j* = 1,…, *m*).

**Step 3 (identify the linguistic variables).**

Identify the appropriate linguistic variables for the importance weight of criteria and the rating for alternatives with regard to each criterion. The decision-makers used TFN linguistic variables shown in Tables 1 and 2 to evaluate the importance of the criteria and the performance ratings of alternatives with respect to qualitative criteria.

**Step 4 (construct the performance rating matrix).**

A typical fuzzy MCDM problem is formally expressed in matrix format as ${\tilde{X}}_{k}=\left\|{\tilde{x}}_{ijk}\right\|$, where ${\tilde{x}}_{ijk}$ is the fuzzy performance rating of alternative *A*_*i*_ with respect to criterion *C*_*j*_ evaluated by *k*th decision-maker DM_*k*_. ${\tilde{x}}_{ijk}=(x_{ijk}^{l},x_{ijk}^{m},x_{ijk}^{u})$ is a linguistic variable denoted by TFNs.

**Step 5 (calculate aggregate fuzzy ratings for the alternatives).**

The aggregated fuzzy performance rating ${\tilde{x}}_{ijk}=(x_{ijk}^{l},x_{ijk}^{m},x_{ijk}^{u})$ of each alternative can be calculated as follows:

(24) xijl=1K∑k=1Kxijkl,xijm=1K∑k=1Kxijkm,xiju=1K∑k=1Kxijku.

**Step 6 (determine the fuzzy best value and the fuzzy worst value of all criteria).**

The fuzzy best value and the fuzzy worst value are determined, respectively, as

(25) x~j+=maxi=1,…,nx~ij,for  benefit  criterion,mini=1,…,nx~ij,for  cost  criterion,

(26) x~j−=mini=1,…,nx~ij,for  benefit  criterion,maxi=1,…,nx~ij,for  cost  criterion,j=1,…,m,

where ${\tilde{x}}_{j}^{+}$ is the fuzzy positive-ideal solution and ${\tilde{x}}_{j}^{-}$ is the fuzzy negative-ideal solution for *j*th criteria.

**Step 7 (calculate the utility and the regret measures).**

The VIKOR ranking implies that the preferred alternative is proximate to the ideal solution, starting from *L* ^*p*^-metric used as an aggregating function in a compromise programming method as follows:

(27) Lip=∑j=1mw~jx~j+−x~ijx~j+−x~j−p1/p,1≤p≤∞;  i=1,…,n.

In the VIKOR method, *L*_*i*_^*p*=1^ as ${\tilde{S}}_{i}$ and *L*_*i*_^*p*=*∞*^ as ${\tilde{R}}_{i}$ are used to formulate the ranking measure. Consider

(28) S~i=∑j=1mw~jx~j+−x~ijx~j+−x~j−,

(29) R~iV=maxj=1,…,m⁡w~jx~j+−x~ijx~j+−x~j−,i=1,…,n,

where ${\tilde{w}}_{j}$ are the weights of criteria, expressing the decision-makers' preference as the relative importance of the criteria, and ${\tilde{S}}_{i}$ and ${\tilde{R}}_{i}$ represent the utility measure and the regret measure. ${\tilde{S}}_{i}$ is shown as the average gap; ${\tilde{R}}_{i}$ is shown as maximal gap for improvement priority. ${\tilde{S}}_{i}$ refers to the separation measure of *A*_*i*_ from the positive-ideal solution; ${\tilde{R}}_{i}$ is the separation measure of *A*_*i*_ from the negative-ideal solution. ${\tilde{S}}_{i}$ and ${\tilde{R}}_{i}$ are used to formulate ranking measure.

To calculate the utility measure (${\tilde{S}}_{i}$) and the regret measure (${\tilde{R}}_{i}$), the VIKOR method uses different metrics *L* ^*p*=1^ and *L* ^*p*=*∞*^. On the other hand, from (28) and (29), it follows that for any *i*, ${\tilde{S}}_{i}>{\tilde{R}}_{i}$; that is, this definition imposes a precondition that each alternative will be close to the negative-ideal solution compared to the positive-ideal solution. As is known it cannot describe the real situation. Consequently, in this study, to formulate the ranking instead of (29), we propose the following measure as separation distance of alternative from negative-ideal solution:

(30) $$\begin{array}{c} {\tilde{R}}_{i}^{MV}=\overset{m}{\underset{j=1}{\sum }}\left|\;\frac{{\tilde{w}}_{j}\left({\tilde{x}}_{ij}-{\tilde{x}}_{j}^{-}\right)}{\left({\tilde{x}}_{j}^{+}-{\tilde{x}}_{j}^{-}\right)}\;\right|,\;i=1,\ldots ,n. \end{array}$$

**Step 8 (compute the values ${\tilde{Q}}_{i}$). **

For ranking the results, we use the following VIKOR index ${\tilde{Q}}_{i}^{V}$ and modified VIKOR index ${\tilde{Q}}_{i}^{MV}$:

(31) Q~iV=λS~i−S~−S~+−S~−+1−λR~iV−R~V−R~V+−R~V−,i=1,…,n,

(32) Q~iMV=λS~i−S~−S~+−S~−+1−λR~iMV−R~MV−R~MV+−R~MV−,i=1,…,n,

where

(33) S~+=maxi=1,…,n⁡S~i,S~−=mini=1,…,n⁡S~i,R~V+=maxi=1,…,n⁡R~iV,R~V−=mini=1,…,n⁡R~iV,R~MV+=maxi=1,…,n⁡R~iMV,R~MV−=mini=1,…,n⁡R~iMV.

The solution obtained by ${\tilde{S}}^{+}$ belongs to a maximum group utility (“majority” rule), the solution obtained by ${\tilde{R}}^{+}$ belongs to a minimum individual regret of the “opponent,” and *λ* ∈ [0,1] is the weight of the decision-making strategy “the majority of criteria” (or “the maximum group utility”). Generally, *λ* = 0.5, which can be adjusted depending on the case; *λ* = 1 indicates that only the average gap is considered, and *λ* = 0 indicates that only the maximum gap is prioritized for improvement. The compromise final solution can be investigated and then selected with “voting by majority” (*λ* > 0.5), with “consensus” (*λ* = 0.5) and with “veto” (*λ* < 0.5). To evaluate VIKOR stability, it is helpful to arrange ranking orders according to the three different values of *λ*. Yazdani and Payam [56] showed that the ranking scores produced by different values of *λ* in VIKOR are very close to each other which mean that the ranking based on values of *λ* does not affect the rank of the best choice.

After simple transformations, from (31) can be expressed as (34):

(34) Q~iVλS~+−S~−S~i+1−λR~V+−R~V−R~iV+λR~V+R~V+−R~V−−S~−S~+−S~−−R~V+R~V+−R~V−=α1S~i+α2R~iV+Δ,

where

(35) α1=λS~+−S~−,α2=1−λR~V+−R~V−,Δ=λR~V+R~V+−R~V−−S~−S~+−S~−−R~V+R~V+−R~V−.

From (35), we observe that for any *i* (*i* = 1,…, *n*) Δ = const, *α*_1_ = const, and *α*_2_ = const. Thus, we conclude that ${\tilde{Q}}_{i}^{V}$ is the linear combination of ${\tilde{S}}_{i}$ and ${\tilde{R}}_{i}$. Therefore, to obtain compromise ranking instead of (31) and (32), we can use the following indices, defined as a linear combination of the measures ${\tilde{S}}_{i}$ and ${\tilde{R}}_{i}$:

(36) Q~iLV=λ·S~i+1−λ·R~iV,

(37) Q~iLMV=λ·S~i+1−λ·R~iMV.

Analogously to TOPSIS method [29], to rank the alternatives, the following indices also will be used:

(38) Q~iTV=S~iS~i+R~iV,

(39) Q~iTMV=S~iS~i+R~iMV.

It is easy to see that, from a computational complexity point of view, the presented modified VIKOR models (36)–(39) are more effective than the original fuzzy VIKOR model (31).

**Step 9 (defuzzification).**

Using (23), defuzzify TFNs ${\tilde{S}}_{i}=(S_{i}^{l},S_{i}^{m},S_{i}^{u})$, ${\tilde{R}}_{i}=(R_{i}^{l},R_{i}^{m},R_{i}^{u})$, and ${\tilde{Q}}_{i}=(Q_{i}^{l},Q_{i}^{m},Q_{i}^{u})$ as follows:

(40) Si=Sil+Sim+Siu3;Ri=Ril+Rim+Riu3;Qi=Qil+Qim+Qiu3.

**Step 10 (rank the alternatives).**

Rank the alternatives, sorting them by the values *Q*, *S*, and *R* in ascending order. The results are six ranking lists. The index *Q*_*i*_ implies the separation measure of *A*_*i*_ from the best alternative. That is, the smaller the value *Q*, the better the alternative.

**Step 11 (propose the compromise solution).**

If the following two conditions are satisfied, then the scheme with a minimum value of $\tilde{Q}$ in ranking is considered the optimal compromise solution according to [57]

(**C1**) * acceptable advantage*: the alternative *A* ^(1)^ has an acceptable advantage, if $(\tilde{Q}\left(A^{\left(2\right)}\right)-\tilde{Q}\left(A^{\left(1\right)}\right))/(\tilde{Q}(A^{\left(n\right)})-\tilde{Q}\left(A^{\left(1\right)}\right))\;\geq 1/\left(n-1\right)$, where *A* ^(1)^ is the best ranked alternative and *A* ^(2)^ is the alternative with second position in the ranking list by the measure $\tilde{Q}$; *n* is the number of alternatives;
(**C2**) * acceptable stability*: the alternative *A* ^(1)^ must also be the best ranked by *S* or/and *R*. The compromise solution is stable within decision-making process, which could be with “voting by majority rule” (when *λ* > 0.5 is needed) or by “consensus” (when *λ* = 0.5) and with “veto” (when *λ* < 0.5).

If one of the conditions is not satisfied, then a set of compromise solutions is proposed, which consists of

(i) alternatives *A* ^(1)^ and *A* ^(2)^ if only condition (**C2**) is not satisfied, or
(ii) alternatives *A* ^(1)^,…, *A* ^(*M*)^ if condition (**C1**) is not satisfied; *A* ^(*M*)^ is determined by the relation $\tilde{Q}(A^{\left(M\right)})-\tilde{Q}(A^{\left(1\right)})\approx 1/(n-1)$ for maximum *M* (the positions of these alternatives are “in closeness”).

## 5. Calculate the Weights of Criteria: Worst-Case Method

The idea of the worst-case method [23] is borrowed from structural system analysis, where the reliability of a system is distributed among its elements according to their ranks. The higher the rank is, the greater the reliability part is. Unlike previous methods [27, 29, 32, 58], where for determination of the weights of criteria has been used technique of paired comparison, this method compares criteria only with the one that is the least important among them.

Let *w*_*j*_^*k*^ be the weight of the criterion *C*_*j*_ given by the decision-maker DM_*k*_ that reflects its importance. Let us suppose that the larger the weight *w*_*j*_^*k*^ of the criterion *C*_*j*_ is, the higher rank its rank *R*_*j*_^*k*^ is. This is formalized by the relation

(41) $$\begin{array}{c} \frac{w_{1}^{k}}{R_{1}^{k}}=\frac{w_{2}^{k}}{R_{2}^{k}}=\cdots =\frac{w_{q}^{k}}{R_{q}^{k}}=\cdots =\frac{w_{m}^{k}}{R_{m}^{k}}. \end{array}$$

Let *w*_*q*_^*k*^ and *R*_*q*_^*k*^ represent the weight and the rank of the least important criterion, respectively, evaluated by the decision-maker DM_*k*_. From (40), we obtain the following expression for the weights of criteria relative to the least important criterion, evaluated by the decision-maker DM_*k*_:

(42) $$\begin{array}{c} w_{1}^{k}=R_{1}^{k}\frac{w_{q}^{k}}{R_{q}^{k}},\\ w_{2}^{k}=R_{2}^{k}\frac{w_{q}^{k}}{R_{q}^{k}},\\ \vdots \\ w_{m}^{k}=R_{m}^{k}\frac{w_{q}^{k}}{R_{q}^{k}},\\ \;k=1,2,\ldots ,K. \end{array}$$

Let us require the following condition to hold:

(43) $$\begin{array}{c} w_{1}^{k}+w_{2}^{k}+\cdots +w_{q}^{k}+\cdots +w_{m}^{k}=1,\;k=1,2,\ldots ,K. \end{array}$$

Substituting (41) into (42), we obtain the weight of the least important criterion. One has

(44) wqk1R1k/Rqk+R2k/Rqk+⋯+Rmk/Rqk=1∑j=1mRjk/Rqk,k=1,2,…,K.

Equations (42) and (44) allow one to calculate the criteria weights using ratios of the ranks of all criteria *C*_*j*_ to the rank of the least important criterion *C*_*q*_. Note that comparison with the least important case guarantees that the condition *R*_*j*_^*k*^/*R*_*q*_^*k*^ ≥ 1 holds for all *j* = 1,2,…, *m* and *k* = 1,2,…, *K*.

In (44), the ratios *R*_*j*_^*k*^/*R*_*q*_^*k*^ of criteria ranks are estimated using Saaty's 1–9 scales [27, 28]. The 1–9 scales are illustrated in Table 1.

From (42) and (44), using (21), we obtain the following aggregated weights of criteria:

(45) $$\begin{array}{c} w_{j}=\frac{1}{K}\overset{K}{\underset{k=1}{\sum }}w_{j}^{k},\;j=1,2,\ldots ,m. \end{array}$$

## 6. An Empirical Study

The purpose of the empirical study is to illustrate the use of the suggested method. The experiment was basically set up upon a real life decision. We have formed an executive committee consisting of five independent decision-makers DM_*k*_ (*k* = 1,…, 5) to choose the best alternative from another five participants (Ph.D. students) (*A*_*i*_, *i* = 1,…, 5) to fill the vacancy in the Training-Innovation Centre of the Institute of Information Technology of Azerbaijan National Academy of Sciences. We have selected five Ph.D. students in different areas (mathematics, linguistics, pedagogy, computer science, and medicine) and three specialists in the fields of information security, pedagogy and educational management, and mathematics from the Training-Innovation Centre of the Institute of Information Technology of Azerbaijan National Academy of Sciences, together with two authors of this paper to set up a creative team to participate in this evaluation.

### 6.1. Formation of the Initial Decision Matrices

As reported above, five decision-makers are asked to evaluate the alternatives with respect to the criteria *C*_1_ ÷ *C*_5_, respectively, using abovementioned linguistic terms reported in Tables 1 and 2.

### 6.2. Aggregation of the Decision Matrices

Table 8 shows the aggregated fuzzy ratings of the alternatives by decision-makers obtained from Tables 3 [Table tab4] [Table tab5] [Table tab6]–7. The aggregated fuzzy rating is calculated using (21).

Moreover positive-ideal (${\tilde{X}}^{+}$) and negative-ideal (${\tilde{X}}^{-}$) solutions are identified. The fuzzy positive-ideal solution (${\tilde{X}}^{+}$) and the fuzzy negative-ideal solution (${\tilde{X}}^{-}$) are calculated using (25) and (26).

### 6.3. Calculation of the Criteria Weights

For calculation of the criteria weights by the worst-case method, the decision-makers independently identified the least important criterion and accordingly its rank. Then, using Saaty's scales, they determined rank of other criteria relative to the least important criterion.  Table 9 represents the ranks of criteria assigned by each decision-maker.

As seen from Table 9 for decision-makers DM_1_, DM_2_, DM_3_, DM_4_, and DM_5_, the least important criteria are *C*_5_, *C*_3_, *C*_2_, *C*_4_, and *C*_1_, respectively. From Table 9, by applying (42) and (44), we obtain the following weights of criteria (Table 10).

All these criteria are benefit criteria. All the calculations were carried out using MS Excel.

### 6.4. Evaluation Results and Discussion

For different values of *λ* = 0.1, 0.5, and 0.9, the values of *S*, *R*, and *Q* are calculated using (28)–(40). Then their fuzzy values, using (23), are defuzzified into crisp values and listed in Tables 11–14. The comparison among the different ranking strategies is also shown in Tables 11–14. In these tables bracket [·] denotes the ranking order.

From Tables 12–14, we observe the following main results:

(i) For all values of *λ*, *A*_3_ is ranked best alternative when the ranking strategies *Q* ^V^ and *Q* ^LV^ are used. And these strategies demonstrate close results for all values of *λ* (closeness will be assessed by Kendal rank correlation below). It is very interesting result! This confirms the rightness of the proposed modification!
(ii) When *λ* = 0.5 and *λ* = 0.9, the *Q* ^V^, *Q* ^LV^, *Q* ^MV^, and *Q* ^LMV^ methods demonstrate close results. And when *λ* = 0.9, ordering of the alternatives by the *Q* ^V^and *Q* ^MV^ methods and, respectively, by the *Q* ^LV^ and *Q* ^LMV^ methods is the same. This result again confirms the rightness of the proposed modification of the VIKOR method.
(iii) For *λ* = 0.1 and *λ* = 0.5  *A*_3_ is ranked as best alternative when the ranking strategies *Q* ^MV^ and *Q* ^LMV^ are used.
(iv) Ordering of the alternatives by the *S*, *Q* ^TMV^, *Q*_*λ*=0.5_^LV^, *Q*_*λ*=0.1_^V^, *Q*_*λ*=0.9_^LV^, and *Q*_*λ*=0.9_^LMV^ methods is the same.

Another interesting result is obtained by averaging the results for different values of *λ*.  Table 15 gives a comparative analysis of the alternatives judged by the average values ${\overset{-}{Q}}^{V}$, ${\overset{-}{Q}}^{LV}$, ${\overset{-}{Q}}^{MV}$, and ${\overset{-}{Q}}^{LMV}$ which are obtained from Tables 12–14 as follows:

(46) Q−V=Qλ=0.1V+Qλ=0.5V+Qλ=0.9V3;Q−LV=Qλ=0.1LV+Qλ=0.5LV+Qλ=0.9LV3;Q−MV=Qλ=0.1MV+Qλ=0.5MV+Qλ=0.9MV3;Q−LMV=Qλ=0.1LMV+Qλ=0.5LMV+Qλ=0.9LMV3.

Looking at Tables 13 and 15, we find that the average values ${\overset{-}{Q}}^{V}$, ${\overset{-}{Q}}^{LV}$, ${\overset{-}{Q}}^{MV}$, and ${\overset{-}{Q}}^{LMV}$ are equal to the values *Q*_*λ*=0.5_^V^, *Q*_*λ*=0.5_^LV^, *Q*_*λ*=0.5_^MV^, and *Q*_*λ*=0.5_^LMV^, respectively. The coincidence of the values ${\overset{-}{Q}}^{LV}$ and *Q*_*λ*=0.5_^V^, ${\overset{-}{Q}}^{LMV}$, and *Q*_*λ*=0.5_^LMV^ is obvious. It can be directly derived from (36). Indeed, let us have *P* values of *Q* for *P* values of *λ*. Consider

(47) $$\begin{array}{c} Q_{p}=\lambda_{p}\cdot S+\left(\;1-\lambda_{p}\;\right)\cdot R,\;p=1,\ldots ,P; \end{array}$$

then the average value $\overset{-}{Q}$ can be calculated as

(48) $$\begin{array}{c} \overset{-}{Q}=S\times \frac{1}{P}\overset{P}{\underset{p=1}{\sum }}\lambda_{p}+R\times \frac{1}{P}\overset{P}{\underset{p=1}{\sum }}\left(\;1-\lambda_{p}\;\right). \end{array}$$

The last expression shows that the average value $\overset{-}{Q}$ is a linear combination of *S* and *R* multiplied by the average values of the parameters *λ*_*p*_ and (1 − *λ*_*p*_), respectively. In this experiment the average value of *λ* is equal to (0.1 + 0.5 + 0.9)/3 = 0.5. Consequently, the ranks of alternatives with respect to the average values ${\overset{-}{Q}}^{LV}$and ${\overset{-}{Q}}^{LMV}$ (Table 15) must coincide with the ranks, corresponding to the values *Q*_*λ*=0.5_^LV^ and *Q*_*λ*=0.5_^LMV^ (Table 13).

Here, an interesting fact is that the average values ${\overset{-}{Q}}^{V}$ and ${\overset{-}{Q}}^{MV}$ are also equal to the values *Q*_*λ*=0.5_^V^ and *Q*_*λ*=0.5_^MV^, respectively. This fact once again confirms rightness of our approach that the simple way computing *Q* is the linear combination of *S* and *R*.

From Table 15, we obtain the final ranking of alternatives obtained by different methods (Table 16).

From Table 16, we see that *A*_3_ is ranked as best alternative when *Q* ^V^, *Q* ^LV^, *Q* ^MV^, *Q* ^LMV^, and *Q* ^TMV^ methods are used, and *A*_2_ is ranked as best alternative when *Q* ^TV^ method is used.

Table 17 demonstrates Kendall rank correlation between different methods [59].

As seen in Table 17, high correlation is obtained between the *Q* ^V^ and *Q* ^LV^ and between the *Q* ^V^ and *Q* ^TMV^ measures.

Checking condition (**C1**) (acceptable advantage),

(49) QTVA2−QTVA1QTVA5−QTVA1=QTVA3−QTVA2QTVA1−QTVA2=1.2970−1.28992.1942−1.2899=0.00710.9043=0.0079<1n−1=15−1=0.25,QTMVA2−QTMVA1QTMVA5−QTMVA1=QTMVA4−QTMVA3QTMVA5−QTMVA3=0.6830−0.60270.9370−0.6027=0.08030.3343=0.2402<0.25;

for *λ* = 0.5,

(50) Qλ=0.5VA2−Qλ=0.5VA1Qλ=0.5VA5−Qλ=0.5VA1=Qλ=0.5VA4−Qλ=0.5VA3Qλ=0.5VA5−Qλ=0.5VA3=0.5508−0.36330.8988−0.3633=0.18750.5355=0.3501≥0.25,Qλ=0.5LVA2−Qλ=0.5LVA1Qλ=0.5LVA5−Qλ=0.5LVA1=Qλ=0.5LVA4−Qλ=0.5LVA3Qλ=0.5LVA5−Qλ=0.5LVA3=0.7180−0.56630.9219−0.5663=0.15170.3556=0.4266≥0.25,Qλ=0.5MVA2−Qλ=0.5MVA1Qλ=0.5MVA5−Qλ=0.5MVA1=Qλ=0.5MVA5−Qλ=0.5MVA3Qλ=0.5MVA4−Qλ=0.5MVA3=0.6061−0.51920.6469−0.5192=0.08620.1277=0.6805≥0.25,Qλ=0.5LMVA2−Qλ=0.5LMVA1Qλ=0.5LMVA5−Qλ=0.5LMVA1=Qλ=0.5LMVA1−Qλ=0.5LMVA3Qλ=0.5LMVA4−Qλ=0.5LMVA3=1.0735−1.03131.1511−1.0313=0.04220.1198=0.3522≥0.25;

for *λ* = 0.1,

(51) Qλ=0.1VA2−Qλ=0.1VA1Qλ=0.1VA5−Qλ=0.1VA1=Qλ=0.1VA4−Qλ=0.1VA3Qλ=0.1VA5−Qλ=0.1VA3=0.6082−0.35100.9581−0.3510=0.25720.6071=0.4237≥0.25,Qλ=0.1LVA2−Qλ=0.1LVA1Qλ=0.1LVA5−Qλ=0.1LVA1=Qλ=0.1LVA2−Qλ=0.1LVA3Qλ=0.1LVA1−Qλ=0.1LVA3=0.5124−0.37230.6999−0.3723=0.14010.3276=0.4277≥0.25,Qλ=0.1MVA2−Qλ=0.1MVA1Qλ=0.1MVA5−Qλ=0.1MVA1=Qλ=0.1MVA2−Qλ=0.1MVA5Qλ=0.1MVA4−Qλ=0.1MVA5=0.4981−0.43130.7812−0.4313=0.06680.3499=0.1909<0.25,Qλ=0.1LMVA2−Qλ=0.1LMVA1Qλ=0.1LMVA5−Qλ=0.1LMVA1=Qλ=0.1LMVA1−Qλ=0.1LMVA5Qλ=0.1LMVA4−Qλ=0.1LMVA5=1.0014−0.97531.3050−0.9753=0.02610.3297=0.0792<0.25;

for *λ* = 0.9,

(52) Qλ=0.9VA2−Qλ=0.9VA1Qλ=0.9VA5−Qλ=0.9VA1=Qλ=0.9VA4−Qλ=0.9VA3Qλ=0.9VA5−Qλ=0.9VA3=0.4934−0.37560.8395−0.3756=0.11780.4639=0.2539≥0.25,Qλ=0.9LVA2−Qλ=0.9LVA1Qλ=0.9LVA5−Qλ=0.9LVA1=Qλ=0.9LVA4−Qλ=0.9LVA3Qλ=0.9LVA5−Qλ=0.9LVA3=0.9106−0.76031.1948−0.7603=0.15030.4345=0.3459≥0.25,Qλ=0.9MVA2−Qλ=0.9MVA1Qλ=0.9MVA2−Qλ=0.9MVA1=Qλ=0.9MVA4−Qλ=0.9MVA3Qλ=0.9MVA5−Qλ=0.9MVA3=0.5126−0.40670.7809−0.4067=0.10590.3742=0.2830≥0.25,Qλ=0.9LMVA2−Qλ=0.9LMVA1Qλ=0.9LMVA5−Qλ=0.9LMVA1=Qλ=0.9LMVA4−Qλ=0.9LMVA3Qλ=0.9LMVA5−Qλ=0.9LMVA3=0.9972−0.85331.2310−0.8533=0.14390.3777=0.3810≥0.25.

We observe that the measures *Q* ^TV^ and *Q* ^TMV^ do not satisfy condition (**C1**), and significant acceptable advantage is obtained by the measure *Q* ^LV^ for all values of *λ*. On the other hand, it can be observed that the measures *Q* ^MV^ and *Q* ^LMV^ are sensitive to the values of *λ*. When *λ* = 0.1, the measures *Q* ^MV^ and *Q* ^LMV^ do not satisfy condition (**C1**); that is, they do not have the acceptable advantage and when *λ* = 0.5 the measure *Q* ^MV^ has the high acceptable advantage (0.6805). Looking at Tables 12–14, we observe sensitivity of the measures *Q* ^MV^ and *Q* ^LMV^ to the values of *λ*. When *λ* = 0.5 and *λ* = 0.9 these measures select *A*_3_ as the best alternative, while when *λ* = 0.1 they select *A*_5_ as the best alternative.

Checking condition (**C2**) (acceptable stability), as can be seen from Table 11, this condition is satisfied; that is, *A*_3_ is ranked as the best alternative by measures *S* and *R*.

## 7. Conclusions

Personnel selection is the process of choosing individuals who match the qualifications required to perform a defined job in the best possible way. Due to its characteristics and capabilities, the fuzzy VIKOR method has been widely studied and applied in personnel selection problem in recent years. The fuzzy VIKOR method focuses on ranking and selecting from a set of alternatives in a fuzzy environment. The fuzzy VIKOR method is based on the aggregating fuzzy measure *Q* that represents distance of an alternative to the ideal solution. In this research, we combined modified fuzzy VIKOR and worst-case approaches to develop a more accurate personnel selection methodology. For an illustrative example, proposed model is conducted on an empirical personnel selection process. In general, according to the results of this study, fuzzy VIKOR is a method which can offer suitable alternative. But when the number of criteria is increased, the process of personnel selection will be very complicated. In comparison to fuzzy VIKOR technique, modified fuzzy VIKOR is easy to handle and more quick method in the process of personnel selection. In addition, the results showed very good agreement between fuzzy VIKOR and modified fuzzy VIKOR methods. The final ranking obtained by the proposed modification of fuzzy VIKOR approach was close in accordance with the ranking obtained by the fuzzy VIKOR method. This indicates the usefulness of the proposed modification.

## Figures and Tables

**Table 1:** Scale of the relative importance of criteria.

Intensity of importance, *R*_*j*_/*R*_*q*_	The relative importance of the criteria *C*_*j*_ and *C*_*q*_	Explanation
1	Equal importance	The criterion *C*_*j*_ is equally important as the criterion *C*_*q*_
2	Weak importance	Intermediate between 1 and 3
3	Moderate importance	The criterion *C*_*j*_ is moderately more important than the criterion *C*_*q*_
4	Moderate more importance	Intermediate between 3 and 5
5	Strong importance	The criterion *C*_*j*_ is strongly more important than the criterion *C*_*q*_
6	Strong more importance	Intermediate between 5 and 7
7	Very strong importance	The criterion *C*_*j*_ is very strongly more important than the criterion *C*_*q*_
8	Very strong more importance	Intermediate between 7 and 9
9	Extreme importance	The criterion *C*_*j*_ is extremely more important than the criterion *C*_*q*_

**Table 2:** Linguistic variables for the performance ratings of alternatives.

Linguistic variables	Corresponding TFNs
Best	(8, 9, 10)
Good	(6, 7, 8)
Fair	(4, 5, 6)
Poor	(2, 3, 4)
Worst	(1, 1, 2)

**Table 3:** Individual fuzzy decision matrix of DM_1_.

Alternatives	Criteria				
	*C*_1_	*C*_2_	*C*_3_	*C*_4_	*C*_5_
*A*_1_	(6, 7, 8)	(4, 5, 6)	(4, 5, 6)	(2, 3, 4)	(6, 7, 8)
*A*_2_	(4, 5, 6)	(8, 9, 10)	(4, 5, 6)	(6, 7, 8)	(6, 7, 8)
*A*_3_	(6, 7, 8)	(6, 7, 8)	(2, 3, 4)	(4, 5, 6)	(8, 9, 10)
*A*_4_	(2, 3, 4)	(1, 1, 2)	(6, 7, 8)	(4, 5, 6)	(4, 5, 6)
*A*_5_	(4, 5, 6)	(2, 3, 4)	(1, 1, 2)	(6, 7, 8)	(4, 5, 6)

**Table 4 tab4:** Individual fuzzy decision matrix of DM_2_.

Alternatives	Criteria				
	*C*_1_	*C*_2_	*C*_3_	*C*_4_	*C*_5_
*A*_1_	(4, 5, 6)	(2, 3, 4)	(1, 1, 2)	(2, 3, 4)	(4, 5, 6)
*A*_2_	(2, 3, 4)	(6, 7, 8)	(2, 3, 4)	(2, 3, 4)	(4, 5, 6)
*A*_3_	(8, 9, 10)	(4, 5, 6)	(6, 7, 8)	(4, 5, 6)	(2, 3, 4)
*A*_4_	(1, 1, 2)	(2, 3, 4)	(4, 5, 6)	(6, 7, 8)	(6, 7, 8)
*A*_5_	(6, 7, 8)	(4, 5, 6)	(2, 3, 4)	(8, 9, 10)	(4, 5, 6)

**Table 5 tab5:** Individual fuzzy decision matrix of DM_3_.

Alternatives	Criteria				
	*C*_1_	*C*_2_	*C*_3_	*C*_4_	*C*_5_
*A*_1_	(8, 9, 10)	(4, 5, 6)	(2, 3, 4)	(4, 5, 6)	(6, 7, 8)
*A*_2_	(6, 7, 8)	(1, 1, 2)	(4, 5, 6)	(2, 3, 4)	(4, 5, 6)
*A*_3_	(2, 3, 4)	(6, 7, 8)	(4, 5, 6)	(1, 1, 2)	(8, 9, 10)
*A*_4_	(4, 5, 6)	(8, 9, 10)	(6, 7, 8)	(6, 7, 8)	(4, 5, 6)
*A*_5_	(2, 3, 4)	(4, 5, 6)	(2, 3, 4)	(8, 9, 10)	(2, 3, 4)

**Table 6 tab6:** Individual fuzzy decision matrix of DM_4_.

Alternatives	Criteria				
	*C*_1_	*C*_2_	*C*_3_	*C*_4_	*C*_5_
*A*_1_	(4, 5, 6)	(2, 3, 4)	(8, 9, 10)	(6, 7, 8)	(4, 5, 6)
*A*_2_	(6, 7, 8)	(2, 3, 4)	(6, 7, 8)	(4, 5, 6)	(2, 3, 4)
*A*_3_	(8, 9, 10)	(6, 7, 8)	(4, 5, 6)	(4, 5, 6)	(4, 5, 6)
*A*_4_	(4, 5, 6)	(8, 9, 10)	(2, 3, 4)	(4, 5, 6)	(6, 7, 8)
*A*_5_	(2, 3, 4)	(4, 5, 6)	(2, 3, 4)	(6, 7, 8)	(8, 9, 10)

**Table 7:** Individual fuzzy decision matrix of DM_5_.

Alternatives	Criteria				
	*C*_1_	*C*_2_	*C*_3_	*C*_4_	*C*_5_
*A*_1_	(6, 7, 8)	(2, 3, 4)	(6, 7, 8)	(4, 5, 6)	(6, 7, 8)
*A*_2_	(2, 3, 4)	(6, 7, 8)	(4, 5, 6)	(1, 1, 2)	(4, 5, 6)
*A*_3_	(8, 9, 10)	(4, 5, 6)	(4, 5, 6)	(6, 7, 8)	(2, 3, 4)
*A*_4_	(1, 1, 2)	(2, 3, 4)	(6, 7, 8)	(4, 5, 6)	(6, 7, 8)
*A*_5_	(6, 7, 8)	(4, 5, 6)	(2, 3, 4)	(6, 7, 8)	(4, 5, 6)

**Table 8:** Aggregated fuzzy performance ratings of the alternatives.

Alternatives	Criteria				
	*C*_1_	*C*_2_	*C*_3_	*C*_4_	*C*_5_
*A*_1_	(5.60, 6.60, 7.60)	(2.80, 3.80, 4.80)	(4.20, 5.00, 6.00)	(3.60, 4.60, 5.60)	(5.20, 6.20, 7.20)
*A*_2_	(4.00, 5.00, 6.00)	(4.60, 5.40, 6.40)	(4.00, 5.00, 6.00)	(3.00, 3.80, 4.80)	(4.00, 5.00, 6.00)
*A*_3_	(6.40, 7.40, 8.40)	(5.20, 6.20, 7.20)	(4.00, 5.00, 6.00)	(3.80, 4.60, 5.60)	(4.80, 5.80, 6.80)
*A*_4_	(2.40, 3.00, 4.00)	(4.20, 5.00, 6.00)	(4.80, 5.80, 6.80)	(4.80, 5.80, 6.80)	(5.20, 6.20, 7.20)
*A*_5_	(4.00, 5.00, 6.00)	(3.60, 4.60, 5.60)	(1.80, 2.60, 3.60)	(6.80, 7.80, 8.80)	(4.40, 5.40, 6.40)
${\tilde{X}}^{+}$	(6.40, 7.40, 8.40)	(5.20, 6.20, 7.20)	(4.80, 5.80, 6.80)	(6.80, 7.80, 8.80)	(5.20, 6.20, 7.20)
${\tilde{X}}^{-}$	(2.40, 3.00, 4.00)	(2.80, 3.80, 4.80)	(1.80, 2.60, 3.60)	(3.00, 3.80, 4.80)	(4.00, 5.00, 6.00)

**Table 9:** The ranks of criteria assigned by each decision maker.

Criteria	The relative importance of the criteria defined by each decision-maker				
	DM_1_	DM_2_	DM_3_	DM_4_	DM_5_
	*R*_*j*_/*R*_5_	*R*_*j*_/*R*_3_	*R*_*j*_/*R*_2_	*R*_*j*_/*R*_4_	*R*_*j*_/*R*_1_
*C*_1_	2	8	5	3	**1**
*C*_2_	6	4	**1**	2	3
*C*_3_	7	**1**	3	6	7
*C*_4_	3	5	8	**1**	2
*C*_5_	**1**	7	6	8	4

**Table 10:** Importance weights of evaluation criteria.

The weights of criteria for each decision-maker (*w*_*j*_^*k*^)					The average weights of criteria (*w*_*j*_)
DM_1_	DM_2_	DM_3_	DM_4_	DM_5_	
*w*_1_^1^ = 0.1053	*w*_1_^2^ = 0.3200	*w*_1_^3^ = 0.2174	*w*_1_^4^ = 0.1500	*w*_1_^5^ = 0.0588	*w*_1_ = 0.1703
*w*_2_^1^ = 0.3158	*w*_2_^2^ = 0.1600	*w*_2_^3^ = 0.0435	*w*_2_^4^ = 0.1000	*w*_2_^5^ = 0.1765	*w*_2_ = 0.1591
*w*_3_^1^ = 0.3684	*w*_3_^2^ = 0.0400	*w*_3_^3^ = 0.1304	*w*_3_^4^ = 0.3000	*w*_3_^5^ = 0.4118	*w*_3_ = 0.2501
*w*_4_^1^ = 0.1579	*w*_4_^2^ = 0.2000	*w*_4_^3^ = 0.3478	*w*_4_^4^ = 0.0500	*w*_4_^5^ = 0.1176	*w*_4_ = 0.1747
*w*_5_^1^ = 0.0526	*w*_5_^2^ = 0.2800	*w*_5_^3^ = 0.2609	*w*_5_^4^ = 0.4000	*w*_5_^5^ = 0.2353	*w*_5_ = 0.2458

**Table 11:** Ranking of alternatives with respect to values of *S*, *R* ^V^, *Q* ^TV^, *Q* ^TMV^, and *Q* ^TMV^.

Alternatives	*S*		*R* ^V^		*R* ^MV^		*Q* ^TV^		*Q* ^TMV^	
	(26)		(27)		(28)		(38)		(39)	
*A*_1_	1.1636	[4]	0.6484	[5]	0.9834	[4]	2.1942	[5]	0.8326	[4]
*A*_2_	1.1038	[3]	0.4467	[2]	1.1005	[3]	**1.2899**	[1]	0.8033	[3]
*A*_3_	**0.8088**	[1]	**0.3238**	[1]	1.2538	[2]	1.2970	[2]	**0.6027**	[1]
*A*_4_	0.9587	[2]	0.4772	[3]	**1.3434**	[1]	1.9910	[4]	0.6830	[2]
*A*_5_	1.2630	[5]	0.5807	[4]	0.9433	[5]	1.6891	[3]	0.9370	[5]

**Table 12:** Ranking of alternatives with respect to values of *Q* ^V^, *Q* ^LV^, *Q* ^MV^, and *Q* ^LMV^ for *λ* = 0.1.

Alternatives	*Q*_*λ*=0.1_^V^		*Q*_*λ*=0.1_^LV^		*Q*_*λ*=0.5_^MV^		*Q*_*λ*=0.5_^LMV^	
	(31)		(36)		(32)		(37)	
*A*_1_	0.8197	[4]	0.6999	[5]	0.6101	[3]	1.0014	[2]
*A*_2_	0.7778	[3]	0.5124	[2]	0.4981	[2]	1.1008	[3]
*A*_3_	**0.3510**	[1]	**0.3723**	[1]	0.6316	[4]	1.2093	[4]
*A*_4_	0.6082	[2]	0.5254	[3]	0.7812	[5]	1.3050	[5]
*A*_5_	0.9581	[5]	0.6489	[4]	**0.4313**	[1]	**0.9753**	[1]

**Table 13:** Ranking of alternatives with respect to values of *Q* ^V^, *Q* ^L+V^, *Q* ^MV^, and *Q* ^LMV^ for *λ* = 0.5.

Alternatives	*Q*_*λ*=0.5_^V^		*Q*_*λ*=0.5_^LV^		*Q*_*λ*=0.5_^MV^		*Q*_*λ*=0.5_^LMV^	
	(31)		(36)		(32)		(37)	
*A*_1_	0.7228	[3]	0.9060	[4]	0.6064	[3]	1.0735	[2]
*A*_2_	0.7783	[4]	0.7753	[3]	0.6229	[4]	1.1021	[3]
*A*_3_	**0.3633**	[1]	**0.5663**	[1]	**0.5192**	[1]	**1.0313**	[1]
*A*_4_	0.5508	[2]	0.7180	[2]	0.6469	[5]	1.1511	[5]
*A*_5_	0.8988	[5]	0.9219	[5]	0.6061	[2]	1.1031	[4]

**Table 14:** Ranking of alternatives with respect to values of *Q* ^V^, *Q* ^LV^, *Q* ^MV^, and *Q* ^LMV^ for *λ* = 0.9.

Alternatives	*Q*_*λ*=0.9_^V^		*Q*_*λ*=0.9_^LV^		*Q*_*λ*=0.9_^MV^		*Q*_*λ*=0.9_^LMV^	
	(31)		(36)		(32)		(37)	
*A*_1_	0.6260	[3]	1.1121	[4]	0.6027	[3]	1.1456	[4]
*A*_2_	0.7788	[4]	1.0381	[3]	0.7477	[4]	1.1034	[3]
*A*_3_	**0.3756**	[1]	**0.7603**	[1]	**0.4067**	[1]	**0.8533**	[1]
*A*_4_	0.4934	[2]	0.9106	[2]	0.5126	[2]	0.9972	[2]
*A*_5_	0.8395	[5]	1.1948	[5]	0.7809	[5]	1.2310	[5]

**Table 15:** Ranking of alternatives with respect to average values of  ${\overset{-}{Q}}^{V}$, ${\overset{-}{Q}}^{LV}$, ${\overset{-}{Q}}^{MV}$, and ${\overset{-}{Q}}^{LMV}$.

Alternatives	${\overset{-}{Q}}^{V}$		${\overset{-}{Q}}^{LV}$		${\overset{-}{Q}}^{MV}$		${\overset{-}{Q}}^{LMV}$	
*A*_1_	0.7228	[3]	0.9060	[4]	0.6064	[3]	1.0735	[2]
*A*_2_	0.7783	[4]	0.7753	[3]	0.6229	[4]	1.1021	[3]
*A*_3_	**0.3633**	[1]	**0.5663**	[1]	**0.5192**	[1]	**1.0313**	[1]
*A*_4_	0.5508	[2]	0.7180	[2]	0.6469	[5]	1.1511	[5]
*A*_5_	0.8988	[5]	0.9219	[5]	0.6061	[2]	1.1031	[4]

**Table 16:** Final ranking.

	${\overset{-}{Q}}^{V}$	${\overset{-}{Q}}^{LV}$	${\overset{-}{Q}}^{MV}$	${\overset{-}{Q}}^{LMV}$	*Q* ^TV^	*Q* ^TMV^
*A*_1_	3	4	3	2	5	4
*A*_2_	4	3	4	3	**1**	3
*A*_3_	**1**	**1**	**1**	**1**	2	**1**
*A*_4_	2	2	5	5	4	2
*A*_5_	5	5	2	4	3	5

**Table 17:** Kendall rank correlation between different methods.

	*Q* ^V^	*Q* ^LV^	*Q* ^MV^	*Q* ^LMV^	*Q* ^TV^	*Q* ^TMV^
*Q* ^V^	1.0	**0.8**	0.0	0.4	0.0	**0.8**
*Q* ^LV^	X	1.0	−0.2	0.2	0.2	1.0
*Q* ^MV^	X	X	1.0	0.6	0.2	−0.2
*Q* ^LMV^	X	X	X	1.0	0.2	0.2
*Q* ^TV^	X	X	X	X	1.0	0.6
*Q* ^TMV^	X	X	X	X	X	1.0
